# Alleviative Effects of a Kampo (a Japanese Herbal) Medicine “Maoto (Ma-Huang-Tang)” on the Early Phase of Influenza Virus Infection and Its Possible Mode of Action

**DOI:** 10.1155/2014/187036

**Published:** 2014-03-20

**Authors:** Takayuki Nagai, Erika Kataoka, Yuka Aoki, Rei Hokari, Hiroaki Kiyohara, Haruki Yamada

**Affiliations:** ^1^Department of Drug Discovery Sciences, Kitasato Institute for Life Sciences, Kitasato University, 5-9-1 Shirokane, Minato-ku, Tokyo 108-8641, Japan; ^2^Graduate School of Infection Control Sciences, Kitasato University, 5-9-1 Shirokane, Minato-ku, Tokyo 108-8641, Japan; ^3^Oriental Medicine Research Center, Kitasato University, 5-9-1 Shirokane, Minato-ku, Tokyo 108-8642, Japan

## Abstract

A Kampo medicine, maoto, has been prescribed in an early phase of influenza-like illness and used for a treatment of influenza clinically in Japan these days. However, the efficacy of maoto against the virus infection remains to be elucidated. This study was conducted to evaluate the alleviative effects of maoto against early phase of influenza virus infection and its preliminary mode of actions through immune systems. When maoto (0.9 and 1.6 g/kg/day) was orally administered to A/J mice on upper respiratory tract infection of influenza virus A/PR/8/34 from 4 hours to 52 hours postinfection (p.i.) significant antipyretic effect was shown in comparison with water-treated control. Administration of maoto (0.8 and 1.3 g/kg/day) significantly decreased the virus titers in both nasal (NLF) and bronchoalveolar lavage fluids (BALF) at 52 hours p.i., and significantly increased the anti-influenza virus IgM, IgA, and IgG_1_ antibody titers in NLF, BALF, and serum, respectively. Maoto also increased significantly the influenza virus-bound IgG_1_ and IgM antibody titers in serum and the virus-bound IgM antibody titer in even the BALF of uninfected A/J mice. These results indicate that maoto exerts antipyretic activity in influenza virus-infected mice and virus reducing effect at an early phase of the infection through probably augmentation of the virus-bound natural antibodies.

## 1. Introduction

Maoto (Ma-Huang-Tang in Chinese) is one of Kampo (traditional Japanese herbal) medicines, composed of 4 medicinal herbs, Ephedrae Herba (stem of* Ephedra sinica* Staph), Cinnamomi Cortex (bark of* Cinnamomum cassia* Blume), Armeniacae Semen (kernel of* Prunus armeniaca* Linné), and Glycyrrhizae Radix (root of* Glycyrrhiza uralensis* Fisher) [1]. Maoto has been used for the treatment of febrile disease, such as influenza-like illness (high fever, headache, pain, and cough) since ancient times, and has been used frequently for a treatment of early phase of recent influenza virus infections in Japan. Recently, maoto has been reported to have antipyretic effect in pediatric patients [2, 3] and adult patients with type A influenza virus infection [4]. However, the anti-influenza virus activity of maoto has not yet been reported.

Among the component herbs of maoto, Ephedrae Herba extract (100–400 *μ*g/mL) has been reported to inhibit the growth of influenza virus A/PR/8/34 (H1N1) by direct addition to the cultures of infected MDCK cells owing to suppression of acidification of cellular compartments such as endosomes and lysosomes essential for the uncoating process of influenza virus [5]. It has also been reported that inhalation of cinnamaldehyde (50 mg/cage), which is contained in Cinnamomi Cortex, suppressed proliferation of influenza virus A/PR/8/34 in the lung of ICR mice [6], and glycyrrhizin, which is a constituent of Glycyrrhizae Radix, has been reported to reduce morbidity and mortality of mice infected with lethal doses of influenza virus A_2_ by intraperitoneal administration (10–40 mg/kg) [7] and to inhibit the growth of influenza virus A/USSR (H1N1) and influenza virus B in embryonated eggs [8, 9]. However, oral* in vivo* anti-influenza virus activities have not been known in these compounds.

Maoto has been used for treatment of early phase of influenza virus infection clinically. Also the upper respiratory tract infection (URTI) of influenza virus has been used as an* in vivo* model of early phase of the virus infection [10, 11]. Therefore, we have studied the alleviative effect of maoto against the early phase of influenza infection using the virus-infected mice by URTI. An intranasal influenza virus infection in A/J mouse was utilized as the model to evaluate the therapeutic efficacy of maoto in the present study. This murine model is the most suitable model for analyzing the efficacy against fever production by URTI with influenza virus A/PR/8/34 among 7 mouse strains. In this study, it has been shown that maoto has an antipyretic activity and virus reducing effect through at least partly augmentations of virus-bound natural antibodies on the early stage of the influenza virus infection when maoto has been used clinically.

## 2. Materials and Methods

### 2.1. Materials

Medicinal plants used for preparation of Maoto, Ephedrae Herba (stem of* Ephedra sinica* Stapf and* Ephedra intermedia* Schrenk et C. A. Meyer) (lot no. US312621, cultivated at Inner Mongolia in China), and Glycyrrhizae Radix (root of* Glycyrrhiza uralensis* Fisher) (lot no. US313018, collected at Chifeng city, Inner Mongolia in China) were purchased from Uchida Wakan-yaku Co. Ltd. (Tokyo, Japan), Cinnamomi Cortex (bark of* Cinnamomum cassia* Blume) (lot no. 002807001, cultivated at Yen Bai province in Vietnam in 2005) from Tochimoto Tenkai-do Co. Ltd. (Osaka, Japan), Armeniacae Semen (kernel of* Prunus armeniaca* Linné) (lot no. 25027031, cultivated at Sichuan province in China in 2004) from Tsumura Co. Ltd. (Tokyo, Japan). A voucher specimen of these plants was deposited at the Laboratory of Biochemical Pharmacology for Phytomedicines, Kitasato Institute for Life Sciences, Kitasato University in Tokyo, Japan. Maoto extract was prepared as follows: mixture of crude drugs consisting of Ephedrae Herba (5.0 g), Cinnamomi Cortex (4.0 g), Armeniacae Semen (5.0 g), and Glycyrrhizae Radix (1.5 g) was decocted with 600 mL of water for 40–50 min to half volume. After the extract was centrifuged at 6000 rpm for 30 min at 20°C, the supernatant was filtrated with filter paper and lyophilized (yield; 13.6%). Oseltamivir phosphate (Tamiflu dry syrup) was purchased from Chugai Pharmaceutical Co. Ltd. (Tokyo, Japan).

### 2.2. Three-Dimensional High Performance Liquid Chromatography (3D-HPLC) Analysis of Maoto Extract

**Figure 1:**
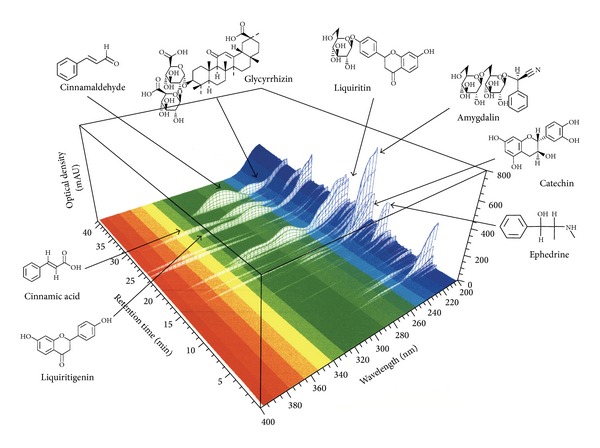
Three-dimensional (3D) HPLC profile of maoto extract.

The 3D-HPLC analysis of maoto extract was performed as previously described [12–14]. The 3D-HPLC profile of an aqueous maoto extract was shown in Figure 1. The analysis based on ultraviolet (UV) absorption clearly showed the presence of the following major constituents in maoto extract: liquiritin, liquiritigenin, and glycyrrhizin (originating from Glycyrrhizae Radix), cinnamaldehyde and cinnamic acid (Cinnamomi Cortex), catechin and ephedrine (Ephedrae Herba), and amygdalin (Armeniacae Semen).

### 2.3. Cell, Virus, and Vaccine

Madin-Darby canine kidney (MDCK) cells were grown in the Eagle's minimum essential medium (EMEM) containing 10% inactivated fetal bovine serum (FBS), penicillin G (100 units/mL), streptomycin (100 *μ*g/mL), and amphotericin B (2.5 *μ*g/mL) (growth medium) in a humidified atmosphere containing 5% CO_2_ at 37°C. Mouse-adapted influenza virus A/PR/8/34 (H1N1) was kept at the Kitasato Institute for Life Sciences, Kitasato University (Tokyo, Japan). The virus was grown in allantoic cavity of 10-day-old embryonated eggs for 48 hours at 34°C. The allantoic fluid was harvested and centrifuged at 1000 ×g for 20 min, and then the resulting supernatant was stored in small portions at −80°C. Influenza HA vaccine was prepared from mouse-adapted influenza virus A/PR/8/34 by the method of Davenport et al. [15], and biotinylated influenza HA vaccine was prepared with Biotinylation kit (Sulfo-OSu) (Dojindo Laboratories, Kumamoto, Japan) according to manufacturer's instructions.

### 2.4. Animals

Specific pathogen-free female DBA/2J, ICR, C57BL/6J, and BALB/cA mice (6 weeks old) were purchased from CLEA Japan, Inc. (Tokyo, Japan), and A/JJms, C3H/HeJYok and ddY mice (6 weeks old) were obtained from Japan SLC, Inc. (Hamamatsu, Shizuoka, Japan). The animals were housed plastic cages in an air-conditioned room at 23 ± 2°C with a relative humidity of 55 ± 10% under a 12-hour light/dark cycle, fed a standard laboratory diet and given water* ad libitum*. Animal experiments were approved by the Institutional Animal Care and Use Committee for Kitasato University and performed in accordance with Guide for the Care and Use of Laboratory Animals in the Kitasato University and the National Research Council Guide for the Care and Use of Laboratory Animals in Japan.

### 2.5. Assay of* In Vitro* Anti-Influenza Virus Activity

Influenza virus A/PR/8/34 at a multiplicity of infection (MOI) of 0.0001 plaque-forming units (PFU)/cell was prepared in infection medium (EMEM, 0.2% bovine serum albumin (BSA), 0.1% glucose, gentamicin (50 *μ*g/mL), and amphotericin B (2.5 *μ*g/mL)) containing acetyltrypsin (3 *μ*g/mL) (Sigma-Aldrich, St. Louis, MO, USA), 180 *μ*L of each dilution was added to the confluent monolayers of MDCK cells in the wells of a 96-well culture plate (Falcon, Becton Dickinson, Franklin Lakes, NJ, USA), and maoto extract dissolved in water (0.25–4 mg/mL) and oseltamivir in water (6.3–100 *μ*g/mL) were added to the wells (20 *μ*L/well). The cells were cultured at 37°C under 5% CO_2_. Three days postinfection (p.i.), the monolayers in the culture plate were separated from the medium and washed with PBS to remove the dead cells resulting from infection with the virus, and the viable cells were determined by a colorimetric method which is based on the* in situ* reduction of 3-(4,5-dimethyl-2-thiazolyl)-2,5-diphenyl-2H-tetrazolium bromide (MTT) (Nacalai Tesque Co. Ltd., Kyoto, Japan) by viable cells as described previously [16].

### 2.6. *In Vivo* Assay of Effects on Influenza Virus Infection 

Mice were used at 7 weeks old in each experiment after a week of rearing. Influenza virus was infected by an URTI as described by the method of Nagai and Yamada [10] and Tamura et al. [11]. Mice were anesthetized by an intraperitoneal injection of Nembutal (sodium pentobarbital, Dainippon Sumitomo Pharma Co. Ltd., Osaka, Japan) and then infected intranasally by dropping 1.5 *μ*L of influenza virus suspension in PBS (pH 7.2) containing 0.1% BSA into each nostril (5 × 10^6^ PFU/mouse). Maoto extract (0.9, 4.5, or 9 mg/mL) and oseltamivir (0.01 mg/mL) were dissolved in water, and then the solutions were administered orally to the mouse from feed-water bottle* ad libitum* from 4 hours to 52 hours p.i. Doses of maoto extract and oseltamivir were calculated from the weight difference of feed-water bottle. The rectal temperature was monitored with a BAT-12 Microprobe Thermometer (Physitemp Instruments, Clifton, NJ, USA) at 0–52 hours p.i. At 52 hours p.i., serum samples were prepared from mice by drawing blood from the axillary artery under anesthesia with inhalation of isoflurane (Mylan Seiyaku Co. Ltd., Tokyo, Japan). A bronchoalveolar lavage fluid (BALF) was obtained by inflating 2 mL of PBS containing 0.1% BSA twice into the lung through the trachea, which was separated from the body [12, 17]. A nasal lavage fluid (NLF) was prepared by washing the nasal cavity of the head with 2 mL of PBS containing 0.1% BSA [17]. The BALF and NLF were centrifuged at 1200 ×g for 20 min at 4°C to remove cellular debris. Serum, BALF, and NLF were kept at −80°C until use.

### 2.7. Plaque Assay 

Infectious influenza virus titers were determined with plaque assay according to the modified method described by Sawamura et al. [18] and Kiyohara et al. [19]. Briefly, triplicate culture of confluent monolayers of MDCK cells in the wells of a 6-well culture plate (Falcon, Becton Dickinson) was exposed to 100 *μ*L of NLF and BALF samples in PBS containing 0.1% BSA at 37°C for 30 min. The cells were overlaid with 2 mL of infection medium containing 0.8% agar (Ina Food Industry Co. Ltd., Nagano, Japan), 0.01% DEAE-dextran (Sigma-Aldrich), 0.1% glucose, and 3 *μ*g/mL of acetyltrypsin followed by cultivation at 37°C for 2 days. The cells were overlaid with 1 mL of the above medium containing 0.01% neutral red (Wako Pure Chemical Industries Co. Ltd., Osaka, Japan) and cultured at 37°C for 6 hours to make plaques more visible. The number of plaques was counted after the cultivation.

### 2.8. ELISA for Influenza Virus-Bound and Total Antibody (Ab) Titers

The influenza virus-bound IgA, IgG_1_, and IgM Ab titers were measured by the modified fluorometric reverse (antibody capture) ELISA as described previously [17, 20]. Briefly, the wells of a 96-well ELISA plate (Immulon 4HBX, Thermo Scientific, Rochester, NY, USA) were coated with 100 *μ*L of the anti-mouse IgA, IgG_1_, or IgM (1 *μ*g/mL) in 50 mM carbonate/bicarbonate buffer (pH 9.5) containing BSA (10 *μ*g/mL). The plates were incubated at 37°C for 3 hours. After the solution was removed, the blocking solution, 1% fat-free milk in PBS, was added to the well (300 *μ*L/well) and incubated at 37°C for 1 hour. The plates were washed four times with PBS containing 0.05% Tween 20 (PBST). Serial dilutions of BALF, NLF, or serum with SuperBlock Blocking Buffer (Thermo Fisher Scientific Inc., Rockford, IL, USA) in PBST were added to the wells. After being sealed with adhesive tape, the plates were incubated overnight at room temperature. After washing with PBST, 1 *μ*g/mL of biotinylated influenza HA vaccine in the blocking solution was added to well (100 *μ*L/well). The plates were incubated at room temperature for 1 hour with shaking on a microplate mixer. After washing with PBST, dilutions of streptavidin-*β*-galactosidase conjugate (Invitrogen, Carlsbad, CA, USA) (1 : 1000) with the blocking solution were added (100 *μ*L/well) and incubated at room temperature for 1 hour with shaking. After the final wash, 0.1 mM 4-methylumbelliferyl-*β*-d-galactoside (Sigma) in buffer A (10 mM sodium phosphate buffer, pH 7.0, containing 0.1 M NaCl, 1 mM MgCl_2_ and 0.1% BSA) was added to well (100 *μ*L/well). The plates were sealed with tape and then incubated at 37°C for 2 hours. The enzyme reaction was stopped by the addition of 100 *μ*L of 0.1 M glycine-NaOH (pH 10.3), and the fluorescence of the 4-methylumbelliferone was measured (ex. 355 nm, em. 460 nm) using an Infinite 200 M microplate reader (Tecan, Männendorf, Switzerland). Endpoint titers of Abs bound to influenza virus were expressed as reciprocal log_2_ titers. Total IgA, IgG_1_, and IgM Ab titers were measured by sandwich ELISA. Briefly, the wells of a 96-well ELISA plate (Immulon 4HBX) were coated with 100 *μ*L of the anti-mouse IgA, IgG_1_, or IgM (BD Pharmingen) (5 *μ*g/mL) in 50 mM carbonate/bicarbonate buffer (pH 9.5) containing BSA (10 *μ*g/mL). The plates were incubated at 37°C for 3 hours. After the solution was removed, the blocking solution, 1% fat-free milk in PBS, was added to well (300 *μ*L/well) and incubated at 37°C for 1 hour. The plates were washed 4 times with PBST. BALF, NLF, or serum in 10% SuperBlock Blocking Buffer in PBST (100 *μ*L/well) were added to the wells, and the plates were incubated overnight at room temperature. After washing with PBST, alkaline phosphatase-labeled anti-mouse IgA (Southern Biotech, Birmingham, AL, USA), IgG_1_ (BD Biosciences), or IgM (BD Biosciences) in the blocking solution was added to the well (100 *μ*L/well). The plates were incubated at 37°C for 1 hour. After the final wash,* p*-nitrophenyl phosphate (1 mg/mL, Kanto Chemical Co. Ltd., Tokyo, Japan) in 10% diethanolamine buffer (pH 9.8) was added to the well (150 *μ*L/well). The plates were sealed with tape and then incubated at 37°C. The enzyme reaction was stopped by the addition of 150 *μ*L of 3 M NaOH, and the absorbance of the* p*-nitrophenol was measured (405 nm–492 nm) using an Infinite 200 M microplate reader. Total Ab titers were determined with standard curve.

### 2.9. Neutralization of Infectivity

Neutralization of infectivity of influenza virus was analyzed in a microneutralization assay based on the methods of the Influenza Reference Laboratories at the Centers for Disease Control and Prevention [21]. Briefly, twofold dilution of BALF was mixed with influenza virus A/PR/8/34 (292 PFU/mL) and incubated at 37°C for 60 min before addition to MDCK cell monolayers. Infectivity of the virus was titrated on MDCK cells by plaque assay as described in Section 2.7.

### 2.10. NK Cell Activity and IFN-*α* Concentration

The NK cell activity of mouse splenocytes was assessed using flow cytometry [22] as described previously [14, 17, 23]. The level of target cell lysis was determined using a Cytomics FC500 flow cytometer (Beckman Coulter, Brea, CA, USA), and the NK cell activity was expressed as the percentage of effector cell-specific lysis. Concentrations of IFN-*α* in BALF and nasal wash were determined with mouse IFN-*α* ELISA kit (R&D Systems, Inc., Minneapolis, MN, USA) according to manufacturer's instructions.

### 2.11. Statistical Analysis

All results were presented as mean ± S.E. from the number of experiments indicated. The significance of differences between three experimental groups was analyzed with one-way ANOVA followed by post hoc multiple comparison. The software used was KaleidaGraph Ver.4.0 (Synergy Software, HULINKS Inc., Tokyo, Japan). The significance of differences between two experimental groups was analyzed with an independent* t*-test. A probability (*P*) value <0.05 was considered significant and *P* value <0.1 was considered a tendency of significant.

## 3. Results

### 3.1. Effect of Maoto Extract on Influenza Virus Infection in MDCK Cells

**Figure 2:**
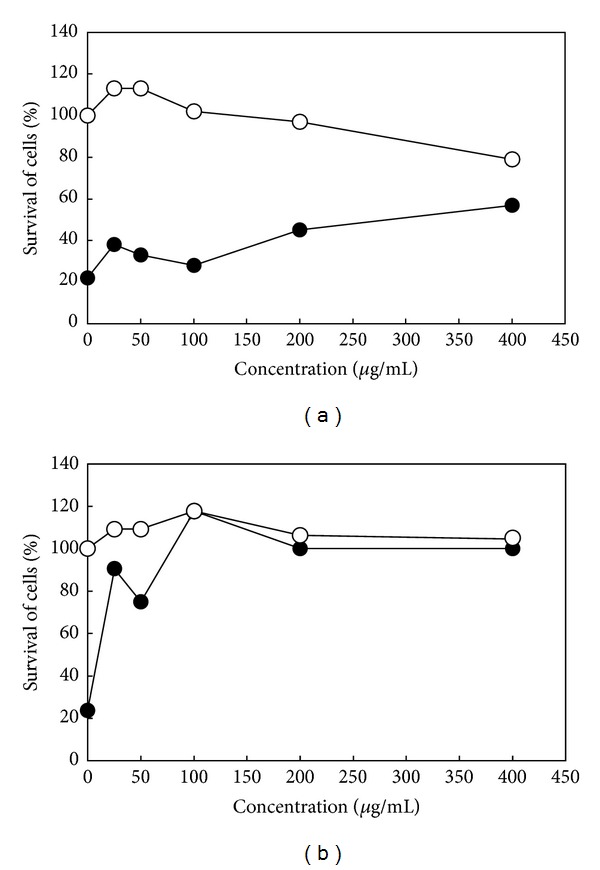
*In vitro* effects of maoto extract (a) and oseltamivir (b) on the survival rate of MDCK cells infected (●) or mock-infected (○) by influenza virus A/PR/8/34.

Antiviral activity of maoto extract against influenza virus A/PR/8/34 (H1N1) in MDCK cells was tested. Maoto extract showed very weak anti-influenza virus A/PR/8/34 activity on the survival of infected MDCK cells (EC_50_≥ 400 *μ*g/mL, Figure 2(a)), although oseltamivir showed a potent antiviral activity against the viral infection with EC_50_ value of 14.3 *μ*g/mL (Figure 2(b)). Maoto extract showed low cytotoxicity for mock-infected MDCK cells at 400 *μ*g/mL (Figure 2(a)).

### 3.2. Fever Production on Upper Respiratory Tract Influenza Virus Infection in Mice

**Figure 3:**
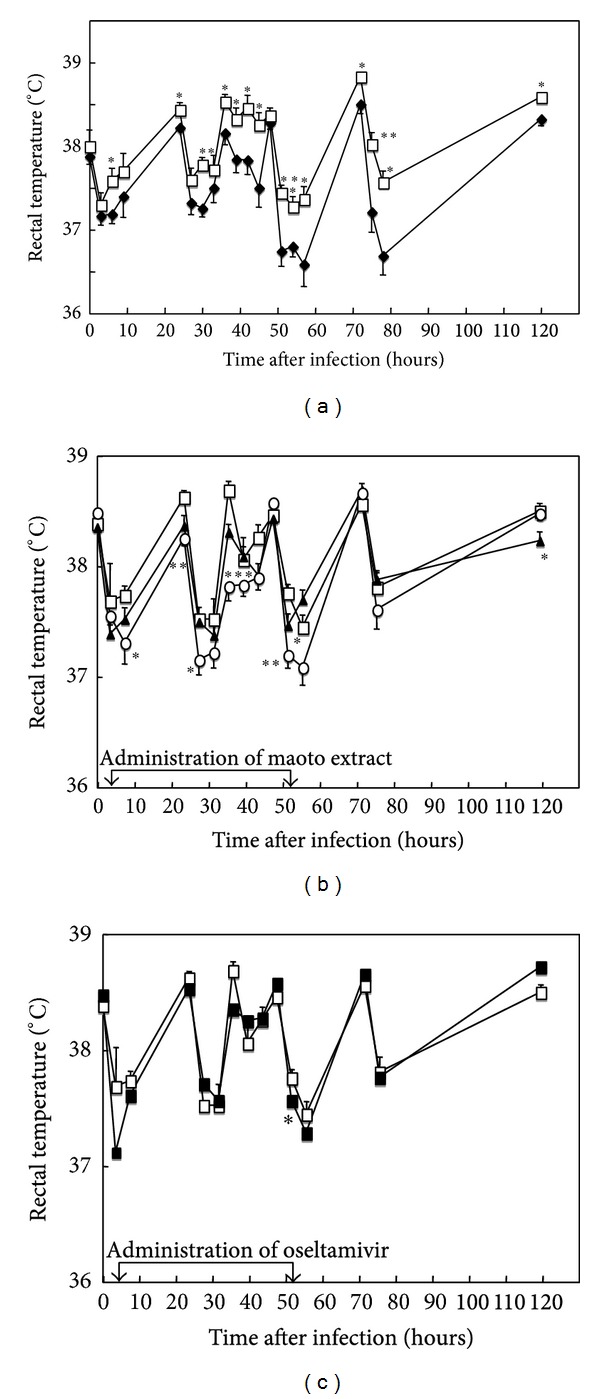
Effects of maoto extract and oseltamivir on the rectal temperature of upper respiratory tract influenza virus-infected mice. A/J mice were mock infected (◆) or infected (□) intranasally with mouse-adapted influenza virus A/PR/8/34, and the rectal temperature was measured for 120 hours (a). The influenza virus-infected mice were administered orally with water (□), maoto extract (0.9 g/kg/day, ▲; 1.6 g/kg/day, ○) (b), or oseltamivir (2.7 mg/kg/day, ■) from 4 to 52 hours postinfection (c). Asterisks indicate significant difference from mock-infected mice (a) or water-administered mice (b, c) (*P < 0.05; **P < 0.01; ***P < 0.001). Values represent mean ± S.E. (*n* = 9).

It has been reported that lower respiratory tract influenza virus infection results in transient fever production in mice [24, 25]. However, the effect of URTI of influenza virus on the fever production in mice has not been studied. Thus fever production was examined in seven strains of mice (DBA/2, ICR, C57BL/6, BALB/c, A/J, C3H/HeJ, and ddY) (female, 7 weeks old) to find a strain with high susceptibility to URTI of influenza virus A/PR/8/34 in fever production. When the rectal temperature was measured in mice after the viral infection, A/J, ddY, C3H/HeJ, ICR, and C57BL/6 strains significantly responded to the virus infection (data not shown). The rectal temperature of A/J mice showed circadian variation, but fever in A/J strain was the most prominent among them that developed from 6 to 120 hours p.i. (Figure 3(a)). Therefore A/J mice were used for the analysis of fever production in this study.

### 3.3. Effect of Maoto Extract on the Rectal Temperature of Influenza Virus-Infected A/J Mice

Mouse-adapted influenza virus A/PR/8/34 (H1N1) was infected to the upper respiratory tract of A/J mice, and maoto extract (0.9 g/kg/day, 1.6 g/kg/day) was administered orally to the mice from 4 to 52 hours p.i. The rectal temperature of maoto extract-treated mice was significantly lower than that of water-treated control at 56 and 120 hours p.i. (0.9 g/kg/day), or 8, 24, 28, 36, and 52 hours p.i. (1.6 g/kg/day) (Figure 3(b)). However, the rectal temperature of oseltamivir (2.7 mg/kg/day) administered mice was significantly lower than that of water-treated control only at 52 hours p.i. (Figure 3(c)). These results suggest that maoto extract (1.6 g/kg/day) showed more potent and quick antipyretic activity than oseltamivir (2.7 mg/kg/day) on the influenza virus A/PR/8/34 infected A/J mice.

### 3.4. Effect of Maoto Extract on the Proliferation of Influenza Virus in the Nasal and Bronchoalveolar Cavities of A/J Mice

**Figure 4:**
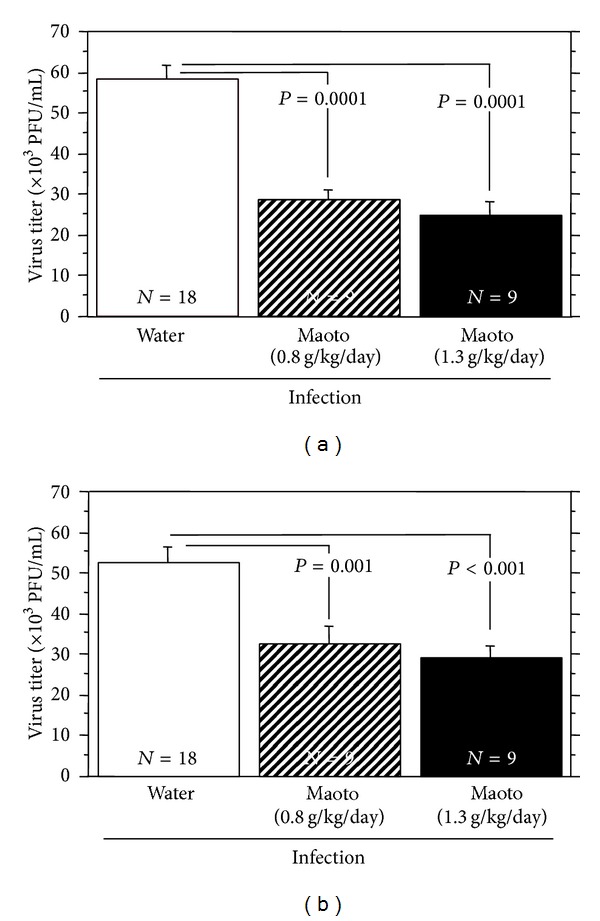
Effect of maoto extract on the proliferation of influenza virus in the nasal and bronchoalveolar cavities of upper respiratory tract influenza virus-infected mice. A/J mice were infected intranasally with mouse-adapted influenza virus A/PR/8/34 and administered orally with water or maoto extract from 4 to 52 hours postinfection. The infectious virus titers in nasal wash (a) and BALF (b) were estimated at 52 hours postinfection with plaque assay. Values represent mean ± S.E. (*n* = 9–18).

When maoto extract was administered orally to A/J mice at doses of 0.8 and 1.3 g/kg/day from 4 to 52 hours p.i., the infectious virus titers in the NLF and BALF at 52 hours p.i. were significantly reduced compared with water-treated group (Figures 4(a) and 4(b)). These results show that oral administration of maoto extract protects the proliferation of influenza virus in the nasal and bronchoalveolar cavities of A/J mice on URTI.

### 3.5. Effect of Maoto Extract on the Influenza Virus-Bound Antibody Titers in Influenza Virus-Infected A/J Mice

**Figure 5:**
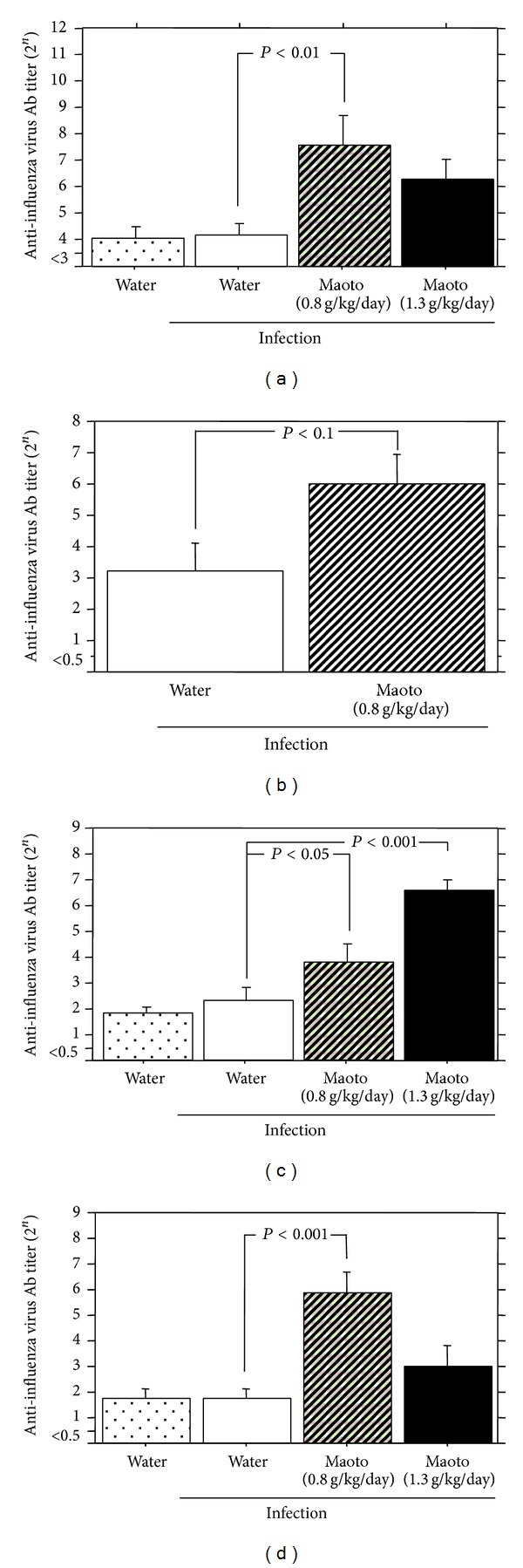
Effect of maoto extract on the influenza virus-bound antibody titers in the upper respiratory tract influenza virus-infected mice. A/J mice were infected intranasally with mouse-adapted influenza virus A/PR/8/34 and administered orally with water or maoto extract from 4 to 52 hours postinfection. Influenza virus-bound IgM (a) and IgA (b) antibody titer in NLF, influenza virus-bound IgA antibody titer in BALF (c), and influenza virus-bound IgG_1_ antibody titer in serum (d) were determined at 52 hours postinfection with ELISA. Values represent mean ± S.E. (*n* = 9).

Influenza virus-bound IgM Ab titer in NLF from the mice treated with maoto extract (0.8 g/kg/day) significantly increased compared with water-treated group at 52 hours p.i. (Figure 5(a)). Administration of maoto extract (0.8 g/kg/day) showed a tendency of increase of virus-bound IgA Ab titer in NLF from the mice (Figure 5(b)). Virus-bound IgA Ab titer in BALF from the mice treated with maoto extract (0.8 and 1.3 g/kg/day) also increased significantly (Figure 5(c)), and virus-bound IgG_1_ Ab titer in serum from the mice treated with maoto extract (0.8 g/kg/day) also significantly increased compared with water-treated group at 52 hours p.i. (Figure 5(d)). These results show that oral administration of maoto extract augments the influenza virus-bound Ab titers in the influenza virus-infected mice even at 52 hours p.i.

### 3.6. Effects of Maoto Extract on Total Antibody Titers in Influenza Virus-Infected A/J Mice

**Figure 6:**
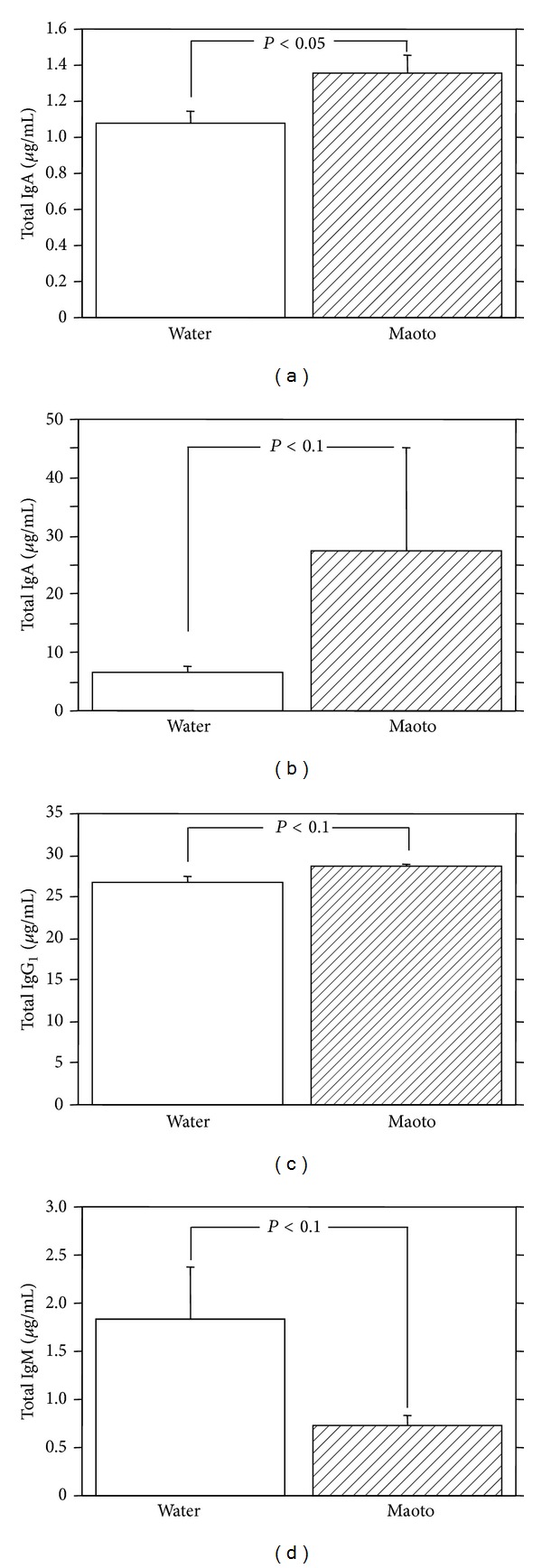
Effect of maoto extract on the total antibody titers in the upper respiratory tract influenza virus-infected mice. A/J mice were infected intranasally with mouse-adapted influenza virus A/PR/8/34 and administered orally with water or maoto extract (0.5 g/kg/day) from 4 to 52 hours postinfection. The total IgA antibody titer in BALF (a) and NLF (b), the total IgG_1_ antibody titer in serum (c), and the total IgM antibody titer in NLF (d) were determined at 52 hours postinfection with ELISA. Values represent mean ± S.E. (*n* = 8).

At 52 hours p.i., the total IgA Ab titer in BALF was increased significantly by oral administration of maoto extract (0.5 g/kg/day) compared to water as a control (Figure 6(a)). The administration of maoto extract showed a tendency of increase of the total IgA Ab titer in NLF and total IgG_1_ Ab titer in serum (Figures 6(b) and 6(c)). Contrary to our expectation, the total IgM Ab titer in NLF was decreased by the administration of maoto extract (Figure 6(d)). These results show that oral administration of maoto extract augments the total IgA Ab titer in respiratory tract and total IgG_1_ Ab in serum of the influenza virus-infected A/J mice but reduces the total IgM Ab titer in nasal cavity.

### 3.7. Effects of Maoto Extract on Influenza Virus-Bound Antibody Titers in Uninfected A/J Mice

**Figure 7:**
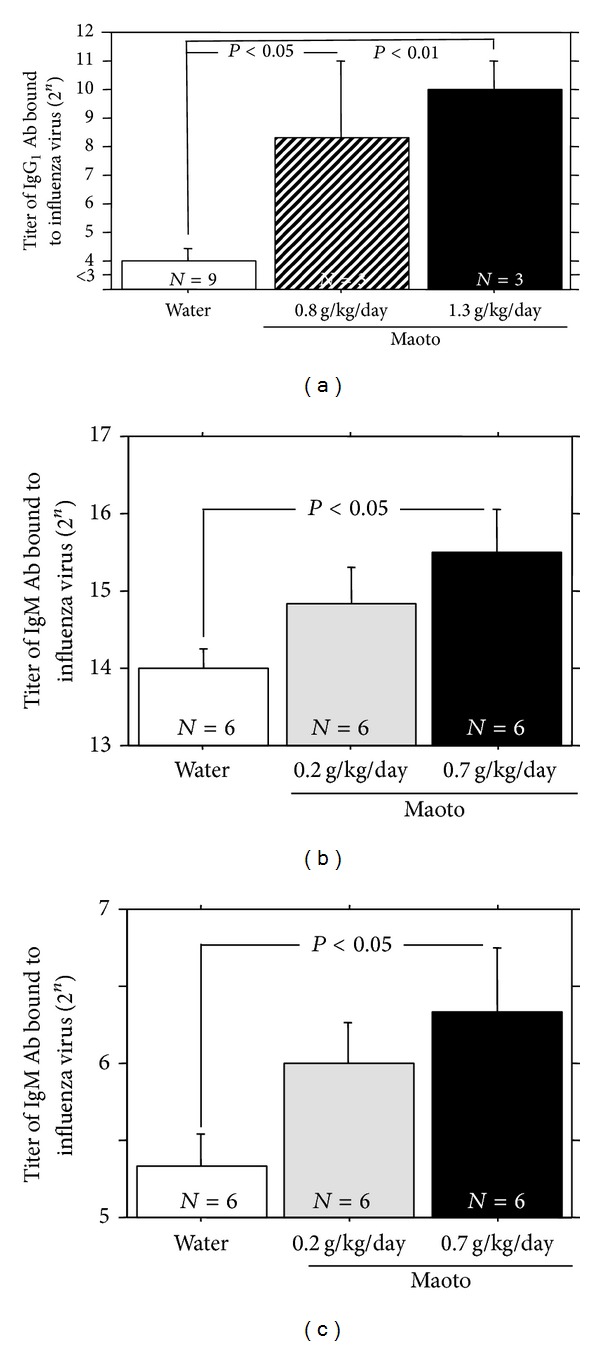
Effect of maoto extract on the influenza virus-bound antibody titers in uninfected mice. Uninfected A/J mice were administered orally with water or maoto extract for 48 hours. The influenza virus-bound IgG_1_ antibody titers in serum (a) and the influenza virus-bound IgM antibody titer in BALF (b) and serum (c) were determined with ELISA. Values represent mean ± S.E. (*n* = 3–9).

**Figure 8:**
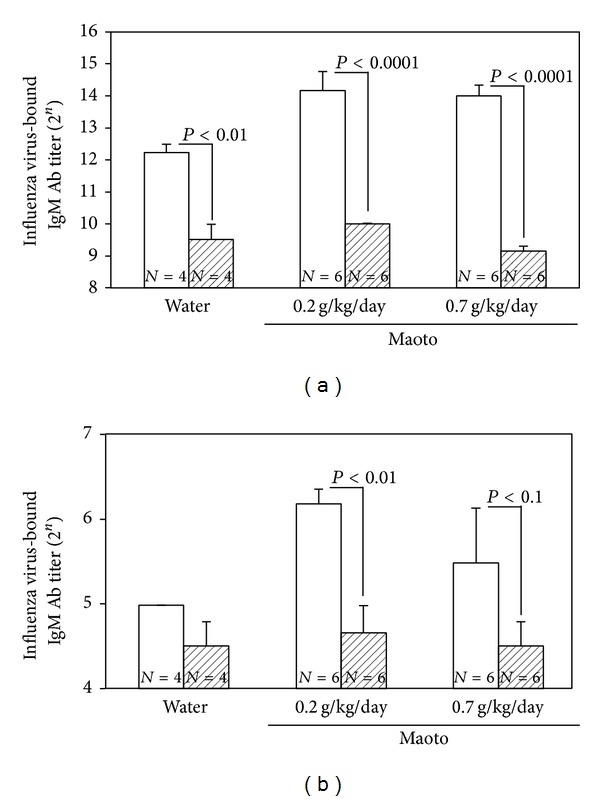
Neutralization of the influenza virus-bound antibody titers in the BALF and serum from uninfected mice by influenza vaccine. The BALF and serum from uninfected A/J mice were incubated with purified influenza A/PR/8/34 vaccine at 4°C overnight and then centrifuged at 10,000 ×g, 4°C for 5 minutes. The influenza virus-bound IgM antibody titers in the supernatant from serum (a) and BALF (b) were determined with ELISA. Open columns show the water-administered mice and hatched columns show maoto extract-administered mice. Values represent mean ± S.E. (*n* = 4–6).

**Figure 9:**
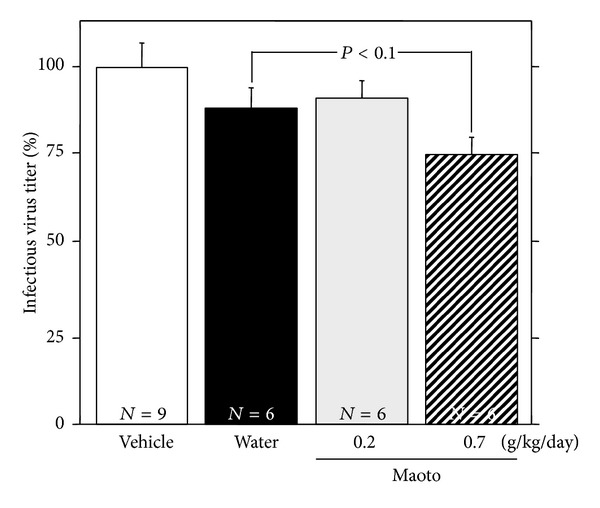
Neutralization effect of BALF from uninfected mice against infectivity of influenza virus A/PR/8/34. Influenza virus A/PR/8/34 was incubated with vehicle (0.1% BSA-PBS) or BALF prepared from water- or maoto extract- (0.2 or 0.7 g/kg/day) administered uninfected mice at 37°C for 60 min, and the infectious virus titers were determined with plaque assay. Values represent mean ± S.E. (*n* = 6–9).

When maoto extract was administered by free oral intake to uninfected A/J mice for 48 hours, influenza virus-bound IgG_1_ Ab titer in serum from the mice treated with maoto extract (0.8 g/kg/day, 1.3 g/kg/day) significantly increased compared with water-treated group (Figure 7(a)). Maoto extract (0.7 g/kg/day) also significantly enhanced the influenza virus-bound IgM Ab titers in the BALF and serum from the uninfected mice (Figures 7(b) and 7(c)). These results show that maoto extract augments the influenza virus-bound Ab titer in the uninfected A/J mice. The serum or BALF prepared from uninfected A/J mice was incubated with influenza HA vaccine at 4°C overnight and then centrifuged at 10,000 ×g for 5 min, and influenza virus-bound IgM Ab titer in the supernatant was measured with ELISA. The influenza virus-bound IgM Ab titers in serum and BALF from maoto extract-administered mice were reduced to the level of water-treated control by the vaccine treatment (Figures 8(a) and 8(b)). These results show that serum and BALF prepared from maoto extract-administered uninfected A/J mice are containing influenza virus-bound IgM Ab. The BALF obtained from maoto extract-administered uninfected mice was incubated with influenza virus A/PR/8/34 at 37°C for 1 hour, and infectious virus titer was measured by plaque assay. The BALF from maoto extract-administered mice showed a tendency of reduction of the infectivity of influenza virus than that from water-administered mice (Figure 9). These results show that the BALF obtained from maoto extract-administered mice can reduce the infectivity of influenza virus more potently than that from water-administered mice, but the neutralizing activity is weak.

### 3.8. Effects of Maoto Extract on NK Cell Activity and IFN-*α* Production

At 52 hours p.i., NK cell activity of splenocytes in maoto-treated group was not increased compared to the water-treated group (data not shown), and the concentration of IFN-*α* in the BALF and NLF of maoto-treated group was not increased compared to the water-treated group (data not shown).

## 4. Discussion

Maoto (Ma-Huang-Tang) is originally recorded in an ancient Chinese medicinal book named “Shang Han Lun (Treatise on Cold Damage Disorders)” and has been historically used in the form of hot-water extract by oral administration and applied for improvement of symptoms in acute phase of febrile diseases, such as influenza virus infection. However, anti-influenza virus activity of maoto has never been elucidated scientifically before the present study. Therefore, antiviral efficacy of maoto on the influenza virus infection was evaluated using MDCK cells* in vitro* and an intranasally influenza virus-infected mice* in vivo* in the present study.

The results demonstrated that maoto extract shows only weak anti-influenza virus activity in MDCK cells at the concentration of 400 *μ*g/mL (Figure 2), suggesting that maoto extract shows negligible direct anti-influenza virus action. Although the extract of Ephedrae Herba, one of the four component herbs of maoto, has been reported to show a direct effect in the influenza virus-infected MDCK cells, but it was effective at relatively high concentration of 100–400 *μ*g/mL [5]. By contrast, oral administration of maoto extract exerted significant alleviative effects against influenza virus infection on mice in the present study. The effect of maoto extract against influenza virus infection in A/J mice was shown as the antipyretic action (Figure 3), decreased virus replication in the bronchoalveolar and nasal cavities (Figure 4), and augmenting influenza virus-bound IgA Ab in BALF, IgG_1_ Ab in serum, and IgM and IgA Abs in NLF (Figure 5) on the virus-infected mice at 52 hours p.i. However, maoto extract did not affect IFN-*α* concentration in BALF, NLF, and NK cell activity in splenocytes of the virus-infected mice (data not shown). These results suggest that maoto extract inhibited the virus growth in the respiratory tract of the host via promoting the humoral and mucosal influenza virus-bound Abs productions.

It has been reported that maoto had a better reducing effect for the duration of fever in children infected with type A influenza [2, 3]. It has also been reported that the duration of fever in maoto-treated group was shortened significantly rather than in oseltamivir-treated group in adult patients with influenza [4]. The present study showed that maoto also decreased the body temperature of influenza virus-infected mice (Figure 3(b)). This is a first report that maoto shows influenza virus reducing effect and antipyretic activity in animal experiment, and this result supports the clinical observations of maoto.

Other Kampo medicines, kakkonto (Ge-Gen-Tang in Chinese) and shoseiryuto (Xiao-Qing-Long-Tang in Chinese) which contain three common component herbs of maoto such as Ephedrae Herba, Cinnamomi Cortex, and Glycyrrhizae Radix, have been reported their protective effects against influenza virus infection in mice. Kakkonto was shown to exhibit an antipyretic action in influenza virus-infected DBA/2 mice by the different mode of action from that of aspirin [26]. When effect of kakkonto on fever production was examined in mice infected with influenza virus A/PR/8/34, kakkonto treatment reduced fever significantly at 1 and 2 days p.i. [26]. In the present study, maoto treatment reduced fever significantly from 8 hours p.i. in mice infected with influenza virus A/PR/8/34 (Figure 3(b)). These results suggest that maoto shows antipyretic activity more quickly than kakkonto in the influenza virus-infected mice. Kurokawa et al. have shown that the fever production is induced in influenza virus-infected mice through elevation of IFN activity, determined by the biological assay, followed by IL-1*α* production [25]. Kakkonto has been reported to have no effect on IFN activity in serum but to suppress IL-1*α* level in the BALF, resulting in reduction of fever on influenza virus-infected mice [26]. In the present study, maoto did not affect IFN-*α* concentration in BALF and NLF of influenza virus-infected mice (data not shown) similar to kakkonto. These results suggest that antipyretic activity of maoto was not caused by the antiviral effect through IFN-*α* production in influenza virus-infected mice. Among the component herbs of kakkonto, it has been reported that the ethanol and chloroform-soluble fractions of* Cinnamomum cassia* (Cinnamomi Cortex) and some cinnamyl derivatives in the fractions showed antipyretic activity [27]. Therefore cinnamyl derivatives may be one of antipyretic substances in maoto, because Cinnamomi Cortex is one of the component herbs of maoto. Elucidations of the mechanism of antipyretic activity of maoto and its antipyretic substance(s) are now in progress by our group.

Shoseiryuto has been reported to reduce the proliferation of influenza virus in the respiratory tract of the virus-infected mice through the enhancement of anti-influenza virus Ab production at 5 days p.i. [10, 28–30] but not at 3 days p.i. [10]. In the present study, maoto inhibited the proliferation of influenza virus in the respiratory tract of the virus-infected mice through the enhancement of influenza virus-bound IgM and IgA Ab productions in nasal cavity, IgA Ab production in bronchoalveolar cavity, and IgG_1_ Ab titer in serum at 52 hours p.i. (Figures 4 and 5). These results suggest that maoto is useful for treatment influenza virus infection at an earlier phase of the virus infection than shoseiryuto. The present study showed that total IgA Ab titer in BALF was increased significantly by oral administration of maoto, and maoto extract showed a tendency of increase of the total IgA Ab titer in NLF and total IgG_1_ Ab titer in serum of the influenza virus-infected mice at 52 hours p.i. (Figure 6). This may relate to the initial concentration of total Ab in each sample. The initial concentration of total IgA Ab in BALF was relatively lower than those of other total Abs; therefore, the augmentation activity of maoto for the production of Abs was shown significantly on total IgA Ab in BALF. Even in uninfected mice, maoto also enhanced the influenza virus-bound IgM Ab titer in BALF and serum and influenza virus-bound IgG_1_ Ab titer in serum (Figures 7 and 8). When the BALF obtained from maoto extract-administered mice was incubated with influenza virus, the infectivity of the virus was more reduced than that from control mice (Figure 9). These results suggest that maoto augments the production of nonspecific Abs including influenza virus-bound Ab in mice. B-1 cell is a subclass of B lymphocyte cells distinct from the major conventional B cells (B-2 cells) in development, function, and tissue location. It has been reported that B-1 cells contribute to protect influenza virus infection even prior to any encounter with the virus by generating natural IgM (i.e., protective Abs that are generated constitutively in the absence of antigenic challenge) [31–35]. Therefore, oral administration of maoto may enhance the production of natural IgM Ab including influenza virus-bound IgM Ab from B-1 cells and/or the proliferation of B-1 cells in uninfected mice. It has been reported that peritoneal B-1 cells are precursors generating a significant proportion of the gut IgA plasma cells [36], and the mucosal IgA response to commensal bacteria by B-1 cells is regarded as part of a primitive mechanism that bridges between innate and adaptive immunity in the gut [36, 37]. B-1 cells have been shown to be the major B cell population not only in the peritoneal cavity but also in the pleural cavity of mouse [38–40]. Therefore maoto may stimulate the migration of B-1 cells to regional lymph node of respiratory tracts and/or the proliferation of IgA-producing B-1 cells at the respiratory tracts. The present study also suggests that maoto induced neutralizing Ab against influenza virus infection in BALF (Figure 9). However, the neutralizing activity of BALF from maoto extract-administered and uninfected mice was not so high (Figure 9). It has a possibility that dilution of the influenza virus-bound Ab in the BAL cavity by inflation with PBS containing 0.1% BSA might result in reduction of the activity. Because the present results suggest that maoto induces the natural Abs including autoantibodies, long-term administration of maoto should be avoided. Although it has been reported that B-1 cells produce autoantibody [41, 42], it is necessary to examine the effects of maoto on migration, proliferation, and Ab production of B-1 cells in the following studies. Elucidation of further mechanisms of action of alleviative effects of maoto on the early phase of influenza virus infection is now in progress.

In conclusion, maoto might have potential as an alternative medicine for the treatment of early phase of influenza through its antipyretic activity and virus reducing effect, having different mechanisms of action from currently approved antiviral drugs, for example, oseltamivir, zanamivir, peramivir and laninamivir; those are neuraminidase inhibitors.
